# Correction to: Fully automated body composition analysis in routine CT imaging using 3D semantic segmentation convolutional neural networks

**DOI:** 10.1007/s00330-020-07443-y

**Published:** 2020-11-27

**Authors:** Sven Koitka, Lennard Kroll, Eugen Malamutmann, Arzu Oezcelik, Felix Nensa

**Affiliations:** 1 Institute of Diagnostic and Interventional Radiology and Neuroradiology, University Hospital Essen, Essen, Germany; 2 Department of General, Visceral and Transplantation Surgery, University Hospital Essen, Essen, Germany

**Correction to: European Radiology**

10.1007/s00330-020-07147-3

The original version of this article, published on 18 September 2020, unfortunately contained a mistake. The following correction has therefore been made in the original: The presentation of the second equation in paragraph “Training details” and of Table 2 was incorrect; the corrected equation and table are given below. The original article has been corrected.

**Table 2:** Evaluation for the fivefold cross-validation runs (stated as mean overall runs) and ensemble predictions on the test set. AC, abdominal cavity; B, bones; M, muscle; ST, subcutaneous tissue; TC, thoracic cavity

				Dice score					
Fivefold CV	Model	*n*_*f*_	*n*_*param*_	AC	B	M	ST	TC	Average
	U-Net 3D	16	5.34 M	0.9509	0.9462	0.9266	0.9432	0.8823	0.9299
		32	21.36 M	0.9669	0.9540	0.9379	0.9574	0.9336	0.9500
		64	85.43 M	0.9682	0.9561	0.9403	0.9582	0.9481	0.9542
	Multi-res U-Net 3D	16	5.82 M	0.9589	0.9484	0.9328	0.9531	0.9211	0.9429
		32	21.24 M	0.9680	0.9554	0.9399	0.9596	0.9414	0.9529
		64	85.10 M	0.9692	0.9564	0.9414	0.9605	0.9452	0.9545
Test set	U-Net 3D	16	5.34 M	0.9609	0.9340	0.9229	0.9553	0.9172	0.9381
		32	21.36 M	0.9731	0.9390	0.9309	0.9610	0.9598	0.9528
		64	85.43 M	0.9739	0.9406	0.9316	0.9623	0.9641	0.9545
	Multi-res U-Net 3D	16	5.82 M	0.9667	0.9355	0.9272	0.9593	0.9518	0.9481
		32	21.24 M	0.9736	0.9409	0.9328	0.9627	0.9629	0.9546
		64	85.10 M	0.973	0.9423	0.9334	0.9623	0.9652	0.9553

$$ {\mathbbm{L}}_{\mathrm{Dice}}=1.0-\frac{1}{C-1}\cdot \sum \limits_{c=2}^C\frac{\sum \limits_{n=1}^N2\cdot {\hat{y}}_{c,n}\cdot {y}_{c,n}+\in }{\sum \limits_{n=1}^N{\hat{y}}_{c,n}+{y}_{c,n}+\in } $$

