# Supplementary material for: Fully automated body composition analysis in routine CT imaging using 3D semantic segmentation convolutional neural networks
Source: Eur Radiol. 2020 Sep 18;31(4):1795–804. doi: 10.1007/s00330-020-07147-3 (PMC7979624; doi:10.1007/s00330-020-07147-3)

Appendix

Table. A.1: Relative absolute volume difference (RAVD) metric in percentage for the 5-fold cross-validation models and respective ensemble model complementary to Table 1.
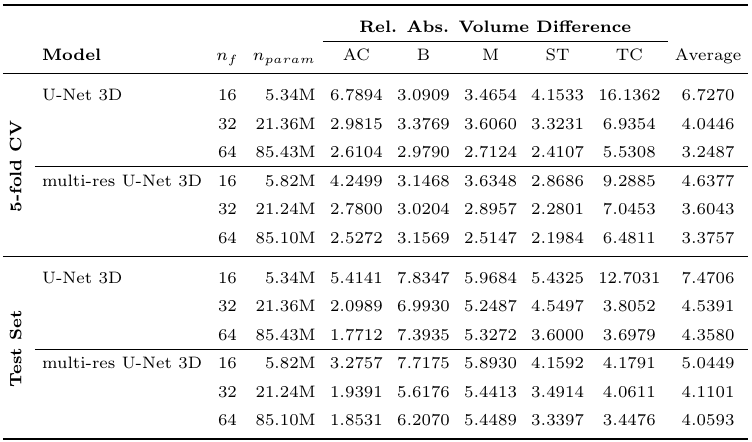


Table A.2: True positive rate (TPR) metric for the 5-fold cross-validation models and respective ensemble model complementary to Table 1.
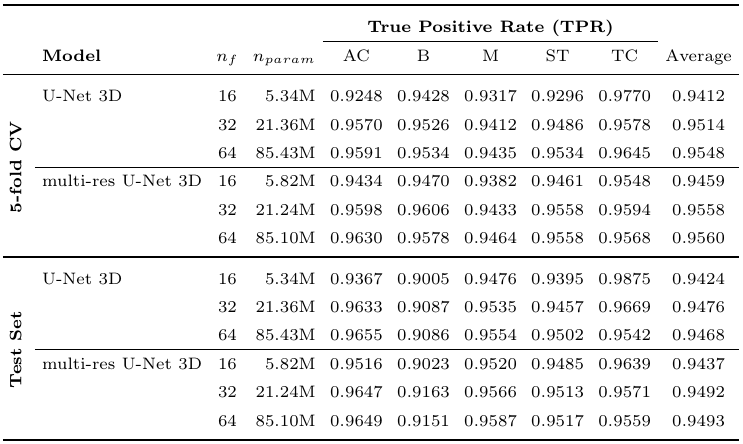


Table A.3: False positive rate (FPR) metric for the 5-fold cross-validation models and respective ensemble model complementary to Table 1.
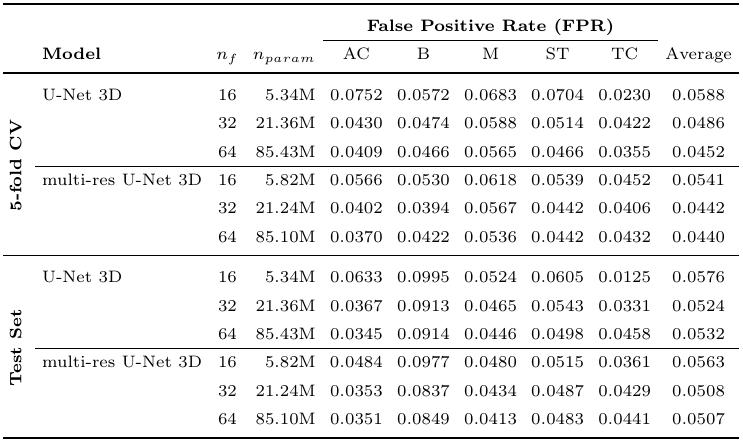

Supplement: Supplementary file 1 — (DOCX 258 kb) [file 330_2020_7147_MOESM1_ESM.docx]
